# Cystic poorly differentiated squamous cell carcinoma of the scalp, a rare scalp tumor: Case report and literature review

**DOI:** 10.1016/j.ijscr.2019.05.055

**Published:** 2019-06-04

**Authors:** Amjad Shah, Jaweria Akram, Abdelrahman Abdelaal, Layth Alateeq, Mohamed Ben-Gashir, Atalla Hammouda

**Affiliations:** a Department of General Surgery, Hamad General Hospital, HMC, Doha, Qatar; b Department of Pathology, Hamad General Hospital, HMC, Doha, Qatar; c Department of Plastic Surgery, Rumailah Hospital, HMC, Doha, Qatar

**Keywords:** SCC, squamous cell carcinoma, MCUP, metastatic carcinoma of an unknown primary site, PTT, proliferating trichilemmal tumor, CPDSCC, cystic poorly differentiated squamous cell carcinoma, Cystic, Squamous cell carcinoma, Trichilemmal carcinoma, Malignant, Trichilemmal carcinoma, Metastatic, Epidermoid cyst, Dermoid

## Abstract

**Introduction:**

Cutaneous cystic lesions have broad differentials ranging from common benign entity to rare malignant lesions. Clinical evaluation of the lesion is the key to differentiation, as some rare malignant entities may simulate benign lesions. A high index of suspicion should be maintained for any aggressive behavior, which may require more thorough evaluation, including histopathology and radiographic imaging studies. We report a rare case of cystic poorly differentiated squamous cell carcinoma (CPDSCC) of the scalp.

**Case presentation:**

We report a case of a 37-year-old Filipino expatriate male who presented with a scalp swelling which had been gradually increasing in size for four months and was operated upon with a working diagnosis of sebaceous cyst. On post-op excisional biopsy, it turned out to be poorly differentiated cystic squamous cell carcinoma of the scalp. On further workup, no metastasis or other primary was found. Complete re-excision of the lesion with no evidence of residual tumor was achieved. Long-term follow-up was lost, as the patient left for his native country.

**Conclusion:**

Though cystic cutaneous lesions are very commonly encountered in clinical practice, high suspicion for malignancy should be maintained if the lesion shows any aggressive behavior. Prompt investigation should be done before surgery to determine the nature of the disease and the most effective management for the patient. Poorly differentiated cystic squamous cell carcinoma should also be considered in the differential diagnosis of cystic cutaneous lesions show aggressive behavior.

## Highlights

• Cutaneous cystic lesion have wide clinical differentials.
• A high index of suspicion should be maintained when any cutaneous lesion shows aggressive behavior.
• A prompt investigation should be carried before surgical resection, and histopathology provides a final diagnosis.
• We report a rare case of cystic poorly differentiated SCC of scalp operated as a sebaceous cyst.

## 1 Introduction

Cutaneous cystic lesions have widely varying clinical differential diagnoses, ranging from a common benign sebaceous cyst to rare metastatic carcinoma of an unknown primary site (MCUP) [1].

Most cystic lesions are diagnosed clinically, but histopathology provides the definitive diagnosis. Sometimes, it is not possible to differentiate a benign from a malignant lesion on clinical basis alone. In such cases, further investigations such as tissue biopsy and radiological examinations are required to narrow down the clinical differential diagnosis.

We are reporting a case of a cystic poorly differentiated squamous cell carcinoma (CPDSCC) of the scalp, to emphasize the importance of keeping a high suspicion of malignancy in dealing with cutaneous cystic lesions, and the importance of preoperative assessment of such lesions.

Every effort has been made to ensure that this work is reported in conformity with SCRAE criteria.

## 2 Case presentation

We report a case of a 37-year-old previously healthy expatriate male, who presented to the emergency department with scalp swelling since four months.

He was referred to surgery out-patient clinic for assessment of the scalp swelling. He presented to Surgery outpatient clinic after a lapse of four months due to unspecified reasons.

The swelling was gradually increasing in size, associated with mild pain. The patient had no history of trauma, fever, weight loss or any other associated symptoms. Past medical, surgical and family history were negative for any malignancy. He was a nonsmoker, nonalcoholic who worked as a laborer.

**Fig. 1:**
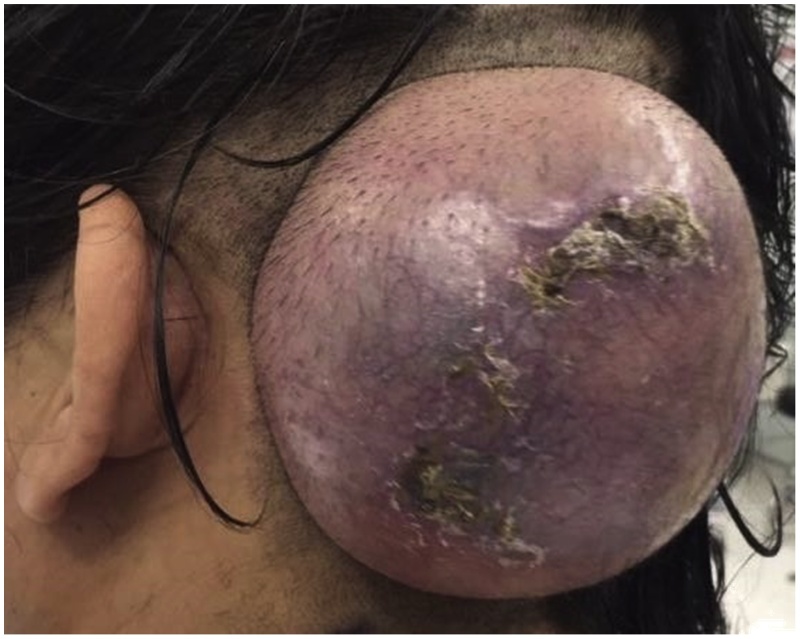
Clinical presentation upon day of surgery.

On examination, there was a solitary scalp swelling (Fig. 1) measuring 7 × 7 cm in size, with a crusted surface. It was a spherical, smooth, subcutaneous lesion on the scalp at the back of the head, 6 cm posterior to left mastoid process. The lesion was tense, freely mobile. It was non-pulsatile, non-compressible, non-reducible and was not trans-illuminating. There was no clinically evident lymphadenopathy. Neck and throat examination was unremarkable. No other abnormal findings were identified.

The provisional diagnosis of sebaceous cyst was made with differentiation diagnoses of dermoid cyst or scalp lipoma.

**Fig. 2:**
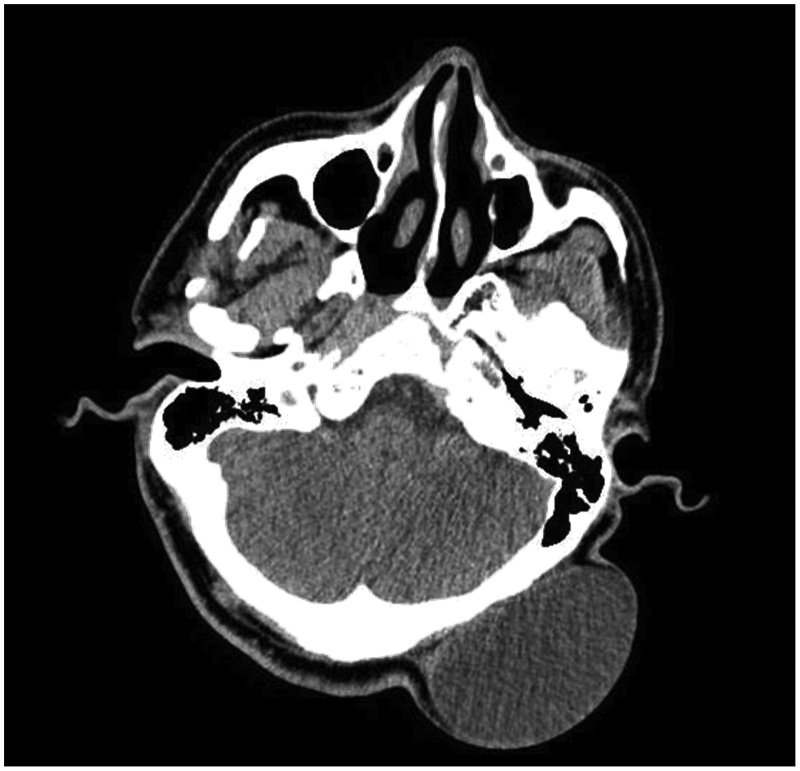
CT head showing subcutaneous extra cranial cystic lesion with no intracranial connections.

Urgent CT scan head was done to rule out any intracranial extension as seen in cases of dermoid cysts, which showed 7 × 3.6 × 7.7 cm subcutaneous extracranial cystic lesion with no intracranial extension (Fig. 2).

The swelling was increasing in size with passage of time of almost eight months from the initial detection by the patient. The possibility of primary malignant lesion or malignant transformation of a benign cyst would have been one of the differentials. The patient’s delayed presentation first to ED and then to surgery OPD prompted the surgery team as crucial factor in posting the patient for urgent excision. Nevertheless, the possibility of malignant lesion was kept in mind during the operation.

Intraoperatively, the patient was found to have a left parietooccipital extracranial scalp lesion with both cystic and soft tissue components, infiltrating the galea aponeurotica and surrounding muscles. Efforts were made for complete excision of the mass, and primary closure was done.

**Fig. 3:**
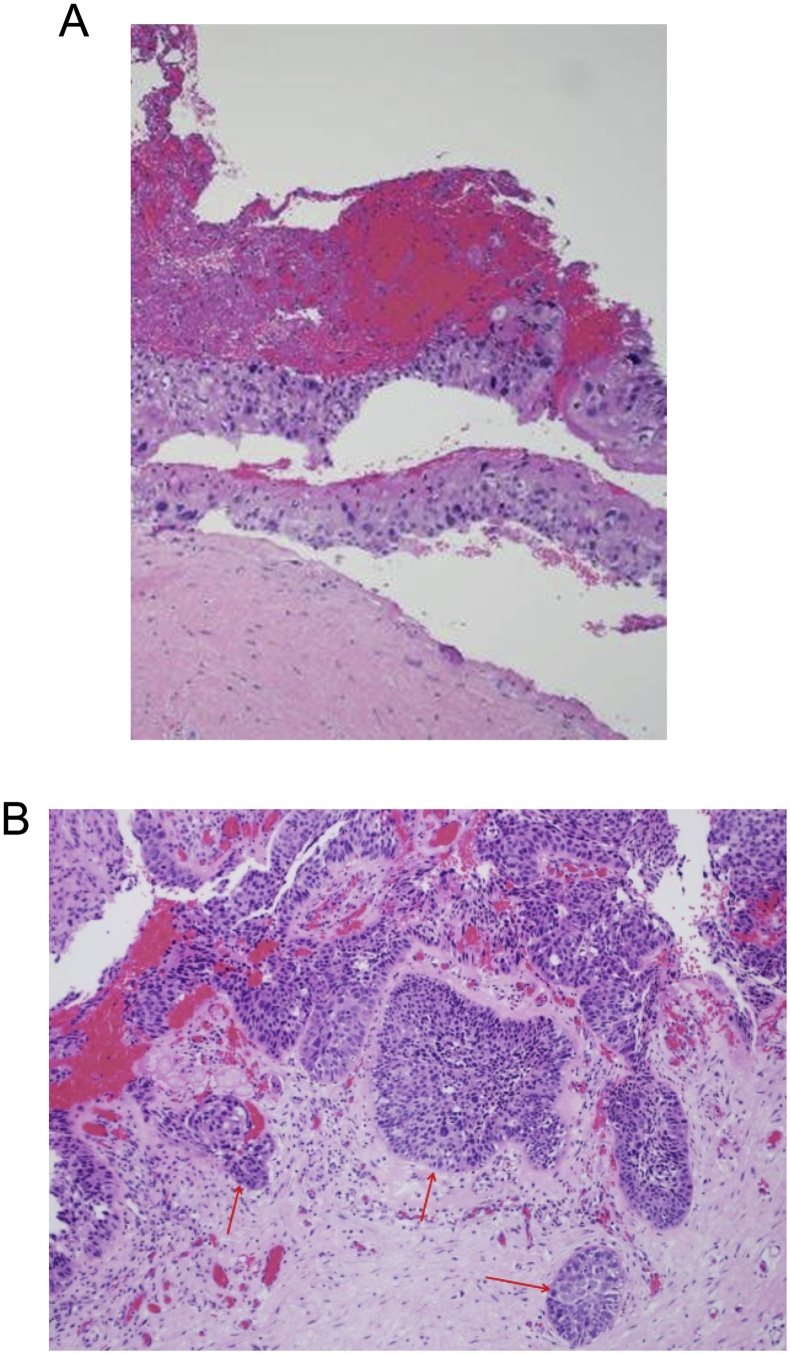
A. H&E section H&E section (2×) : Low power of the cyst lined by malignant squamous epithelium. B. H&E section H&E section (10×) High power of cyst lining with foci of stromal invasion. (highlighted by red arrow) by islands of malignant squamous epithelium.

The entire specimen was sent for histopathological examination. The specimen comprised of several fragments of tissue and pieces of skin. The sections showed fragments of cyst wall lined by malignant squamous epithelium with no benign epithelial component identified (Fig. 3A–C). Foci of dermal invasion with desmoplastic stroma reaction were seen. The histopathological differential diagnosis included a CPDSCC, malignant PTT and trichilemmal carcinoma. Given the poorly differentiated nature of the epithelium and lack of a benign cyst component, the diagnosis of cystic poorly differentiated squamous carcinoma was favored.

The patient was discussed in skin multidisciplinary team (MDT) meeting. PET scan and neck ultrasound were performed to examine for disease extent.

**Fig. 4:**
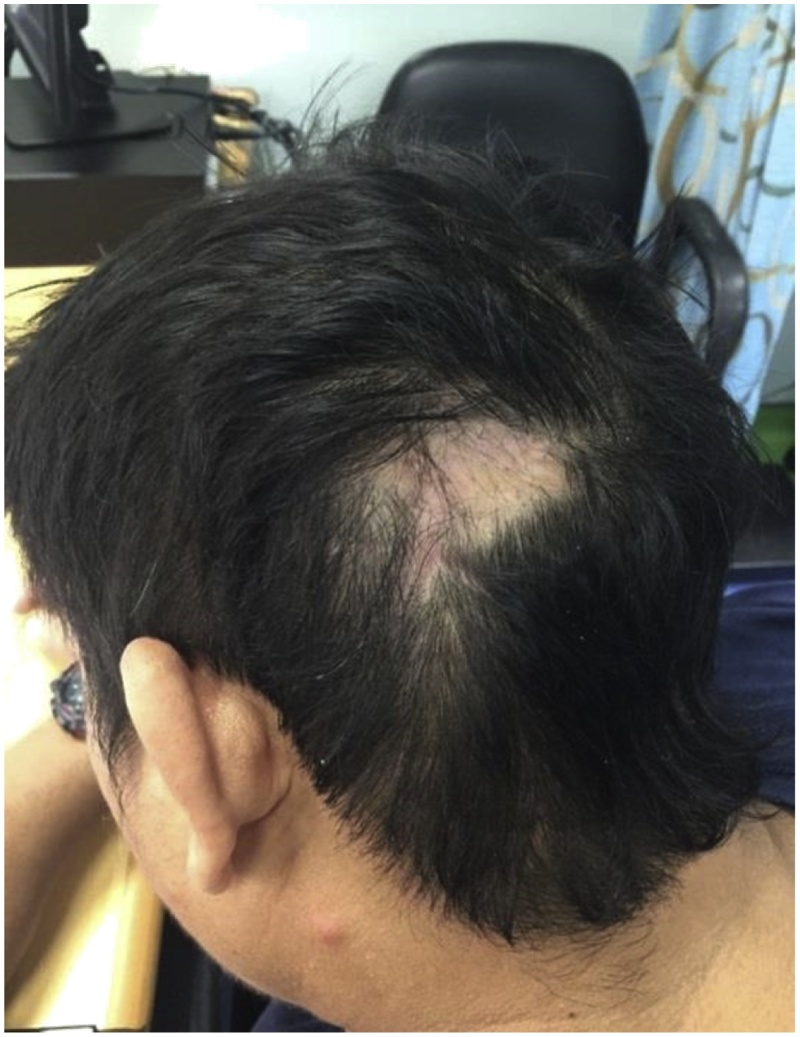
Patient’s presentation on follow up after 2nd surgery.

Re-excision and reconstruction were performed after a negative PET scan and ultrasound of the neck. The histopathology showed no residual tumor. Post-op, the patient did well (Fig. 4).

Long-term follow-up was not possible, as the patient left for his native country.

## 3 Discussion

Cutaneous cystic lesions are very common, and seen in routine daily practice. They have a wide range of differentiators, from common benign lesions to rare malignant lesions. Most of the benign lesions are diagnosed by clinical judgment that is confirmed in the majority of the cases by post-operative histopathological examination. However, in rare instances a malignant lesion which was initially thought to be benign is diagnosed histologically after surgery. Therefore, clinical suspicion of malignancy should be maintained in cases where the clinical assessment shows suspicious features or aggressive behavior such as rapid growth, large size at presentation, fixation to surrounding structures and ulceration. This will require a prompt evaluation, which should include detailed clinical history, local and systemic examination. Further investigations such as CT, MRI or PET scan can be ordered depending on the initial clinical findings, to assess the extent of local disease and to rule out distant metastasis. Fine-needle aspiration and tissue biopsy should also be considered when the clinical suspicion of malignancy is high. Excisional biopsy should be performed when the nature of the lesion cannot be ascertained on the initial clinical assessment. These measures must be implemented to establish the correct diagnosis and decide on the best way to manage the patient.

In our case, an initially begin-looking lesion with a provisional diagnosis of sebaceous cyst turned out to be rare CPDSCC which can either be primary or MCUP [1]. The differential diagnoses included sebaceous cyst, dermoid cyst or scalp lipoma on clinical judgment, and trichilemmal carcinoma or malignant PTT by histopathology.

Epidermoid cysts or epidermal inclusion cysts or inappropriately sebaceous cysts are among the most commonly occurring cutaneous cysts. The diagnosis is usually clinical; they appear as a cystic or nodular lesion, and cysts larger than 5 cm are rare. Histology can confirm the diagnosis. Malignant transformation can occur, although it is rare; but transformation into basal cell carcinoma, SCC [2], melanoma, Bowen's disease or Paget disease have been reported. Based on 13 reported cases, Anton Badiola et al. found that mean age at presentation of SCC arising from epidermal inclusion cyst was 43.2 years [3]. They also found that it is more common in men, mostly occurring in the head and neck region, and the mean diameter was found to be 5.7 cm. No established risk factor is known, but possible triggers such as sun-related skin damage and human papillomavirus have seen suggested [3].

In the literature, cystic SCC is only described as metastatic cystic SCC of the cervical lymph nodes, with the origin of the primary site being in the upper aerodigestive lymphatic system [4]. In the past, these cystic cancers have often been misdiagnosed as branchiogenic carcinomas; that is, malignant transformation of a branchial cleft cyst. Presently, it has been concluded that so-called branchiogenic carcinomas are cystic metastases in the neck arising from a primary oropharyngeal SCC [4].

Thompson et al. [4] reviewed 136 cases of cervical cystic SCC, which included 28 females and 108 males. All of them presented with neck lesion. The mean age at presentation was 54.2 years, and duration of symptoms was 4.5 months on average. Follow-up was available in all cases. During the follow-up, 87 primaries (63%) were identified at the base of the tongue, the lingual tonsil region or the faucial tonsil region, 11 primaries were in the nasopharynx; 11 primaries were in the larynx, palate or sinuses; and 27 primaries were of unknown location or as yet undiscovered. Primaries were identified from the day of the initial surgery to 11 years later. The average time for discovery of the primary was 12.4 months. These patients underwent surgery alone or in combination with radiotherapy or chemotherapy or both. Statistics for all subgroups were provided.

As most of the primaries were occult and small, and some had stayed undiscovered for several years, random biopsies or tonsillectomy were needed to identify the tumors. The primary tumor appeared histologically similar to that of the metastatic lesion. Thorough physical examination under anesthesia with panendoscopy and comprehensive radiographic studies should be done. This type of carcinoma appears to have a more indolent growth behavior compared with most SCCs. The survival at 5 years was found to be 77%, and it was concluded that with the identification of the primary, patients could be treated relatively conservatively with surgical excision and subsequent field-limited radiation therapy [4].

Regarding the mechanism of formation of cystic SCC, it is postulated that most of these metastatic cystic SCCs to lymph nodes are pseudocysts, although some are true cystic cavities. There is substantial evidence regarding formation of true cystic metastatic SCC to cervical lymph nodes that advocates origin from the salivary ducts [5]. It has been noted that nodal metastases from HPV-related SCCs of the upper aerodigestive tract are prone to be cystic [5,6].

In another study involving 121 adult patients who presented with an initial diagnosis of a lateral cervical cyst, metastatic cystic SCC was demonstrated histologically after surgical excision in 12 patients. The incidence of malignancy was significantly higher in patients over 40 years old. Results of preoperative fine-needle aspiration (FNA) were negative for malignancy in five cases of metastatic SCC. Panendoscopy with directed biopsies revealed an occult primary in the base of the tongue in three patients, tonsil in one patient and nasopharynx in one. No primary was found in six patients, despite repeated examinations and close follow-up [7].

In our case, the cyst was lined by a poorly differentiated squamous epithelium with no specific lineage. Trichilemmal carcinoma had been considered, but there were no specific histological or immunohistochemical features to consolidate this diagnosis. The other diagnosis considered was a malignant proliferating trichilemmal cyst, an entity that is believed to be a variant of SCC, by Ackerman and colleagues [8], a belief that has been disputed by other dermatopathologists and has not received wide acceptance [9]. In view of this and the poorly differentiated nature of the cyst lining, a diagnosis of CPDSCC was made.

This rare variant of SCC, diagnosed as CPDSCC, presented as a scalp lesion rapidly growing in a patient with no established risk factors for SCC. It was excised entirely, with further workup showing localized cystic SCC of the scalp, which may itself be primary or metastatic with unknown/undiscovered primary at the time of diagnosis. There was no distant or local metastasis and no vascular or lymphatic or neural invasion. In view of the literature review some further investigation such as panendoscopy should have been performed, but as the case is of very atypical presentation, the actual course of investigation cannot be established or rationalized. It had no early reoccurrence. But as follow-up was lost, prognosis after the surgical excision could not be established.

## 4 Conclusion

We report a very rare case of CPDSCC of the scalp. It is essential to consider this entity during clinical evaluation of unusual cutaneous cystic lesion.

Any cutaneous cystic lesion should be thoroughly evaluated clinically for malignant features. Those showing suspicious behavior such as rapid growth or large size at presentation should be further investigated with imaging and tissue biopsy. This approach would help in deciding the best management for the patient.

## Conflicts of interest

None.

## Funding

None.

## Ethical approval

Protocol ID MRC-04-18-181, Qatar MRC.

## Consent

Consent from patient.

## Author contribution

Amjad Ali Shah concept, design, collection, interpretation, writing.

Jaweria Akram concept, design, collection, interpretation, writing.

Mohamed Ben-Gashir concept, design, collection, interpretation, writing.

Abdelrahman Abdelaal collection, interpretation, writing.

Atalla Hammouda collection, interpretation, writing.

Layth Alateeq collection, interpretation, writing.

## Registration of research studies

N/A.

## Guarantor

Amjad Ali Shah, corresponding author.

## Provenance and peer review

Not commissioned, externally peer-reviewed.

## Availability of data and material

All data generated or analyzed during this study are included in multiple published articles, the links to which are reported and can be accessed through the references.
